# Salicylate Prevents Virus-Induced Type 1 Diabetes in the BBDR Rat

**DOI:** 10.1371/journal.pone.0078050

**Published:** 2013-10-16

**Authors:** Chaoxing Yang, Agata Jurczyk, Philip diIorio, Elaine Norowski, Michael A. Brehm, Christian W. Grant, Dennis L. Guberski, Dale L. Greiner, Rita Bortell

**Affiliations:** 1 Program in Molecular Medicine, University of Massachusetts Medical School, Worcester, Massachusetts, United States of America; 2 Biomedical Research Models, Worcester, Massachusetts, United States of America; University of Hong Kong, Hong Kong

## Abstract

Epidemiologic and clinical evidence suggests that virus infection plays an important role in human type 1 diabetes pathogenesis. We used the virus-inducible BioBreeding Diabetes Resistant (BBDR) rat to investigate the ability of sodium salicylate, a non-steroidal anti-inflammatory drug (NSAID), to modulate development of type 1 diabetes. BBDR rats treated with Kilham rat virus (KRV) and polyinosinic:polycytidylic acid (pIC, a TLR3 agonist) develop diabetes at nearly 100% incidence by ~2 weeks. We found distinct temporal profiles of the proinflammatory serum cytokines, IL-1β, IL-6, IFN-γ, IL-12, and haptoglobin (an acute phase protein) in KRV+pIC treated rats. Significant elevations of IL-1β and IL-12, coupled with sustained elevations of haptoglobin, were specific to KRV+pIC and not found in rats co-treated with pIC and H1, a non-diabetogenic virus. Salicylate administered concurrently with KRV+pIC inhibited the elevations in IL-1β, IL-6, IFN-γ and haptoglobin almost completely, and reduced IL-12 levels significantly. Salicylate prevented diabetes in a dose-dependent manner, and diabetes-free animals had no evidence of insulitis. Our data support an important role for innate immunity in virus-induced type 1 diabetes pathogenesis. The ability of salicylate to prevent diabetes in this robust animal model demonstrates its potential use to prevent or attenuate human autoimmune diabetes.

## Introduction

Development of human type 1 diabetes requires a susceptible genetic background. However, most patients (~85%) have no family history of the disease and concordance is only ~50% among identical twins, indicating that expressivity of type 1 diabetes susceptibility genes is environmentally dependent [1,2]. Epidemiologic and clinical evidence suggests virus exposure may be a ‘trigger’ for type 1 diabetes development [3,4]. Mechanistic studies of virus-induced diabetes are not feasible in humans, therefore reliable virus-inducible animal models are needed. 

A well-established animal model of virus-inducible type 1 diabetes is the BioBreeding Diabetes Resistant (BBDR) rat. When treated with the parvovirus Kilham rat virus (KRV) and a TLR3 agonist, polyinosinic:polycytidylic acid (pIC), BBDR rats develop diabetes at a high rate (~100%), with consistent kinetics (~2 weeks) [5,6]. This highly reproducible model allows us to dissect the progression of disease from viral infection to onset of hyperglycemia in distinct temporal stages. KRV does not infect pancreatic beta cells directly, but induces autoimmune diabetes through alteration of the immune system [7,8]. Microarray studies of pancreatic lymph nodes from KRV-infected BBDR rats show upregulation of many proinflammatory cytokine genes prior to diabetes onset [9]. In humans, serum levels of proinflammatory cytokines and C-reactive protein (CRP) are elevated in newly-diagnosed type 1 diabetes patients compared to age-matched controls [10], and increased TLR expression or responsiveness of PBMCs from these patients is associated with elevated NF-κB signaling [11,12]. 

Mechanistic studies demonstrate inhibition of NF-κB activity by non-acetylated salicylates, a class of non-steroidal anti-inflammatory drugs (NSAIDs) [13,14]. The TINSAL-T2D (Targeting Inflammation using Salsalate for Type 2 Diabetes) study demonstrated that Salsalate (a prodrug form of salicylate) lowers circulating free fatty acids, resulting in improved glucose and lipid homeostasis [15–17]. These data support therapeutic targeting of inflammation and NF-κB in type 2 diabetes. We hypothesize that salicylates may also target the inflammatory components of type 1 diabetes and may be efficacious in the prevention or treatment of this disease. Here we utilize the virus-inducible BBDR rat to investigate the ability of salicylate to modulate the inflammatory innate immune response and subsequent development of autoimmune type 1 diabetes.

## Materials and Methods

### Animals

BBDR rats obtained from Biomedical Research Models, Inc. were maintained in a viral-antibody-free facility and maintained in accordance with the Guide for the Care and Use of Laboratory Animals (Institute of Laboratory Animal Resources, 1996) and guidelines of the Institutional Animal Care and Use Committee of the University of Massachusetts Medical School (approval number #A-1766). 

### Diabetes induction and salicylate treatment

BBDR rats of both sexes aged 21-24 days were injected intraperitoneally (i.p.) with pIC (Sigma) (1-2 µg/g body weight, in PBS) for three days (days -3, -2, and -1), followed by a single i.p. dose of 1x10^7^ PFU of KRV (or non-diabetogenic H1 virus) on day 0. Sodium salicylate (SS, in PBS) (Calbiochem) was injected i.p. on the same days as pIC (days -3, -2, and -1) and KRV (day 0). Rats were monitored for glycosuria (Clinistix, Bayer); diabetes was confirmed by blood glucose >250 mg/dL on two consecutive days (Accu-Chek, Roche Diagnostics).

### Pancreatic histology, insulitis scoring, and immunofluorescence staining

Rat pancreata were fixed in 10% buffered formalin and embedded in paraffin. Sections were stained with hematoxylin and eosin (H&E). Insulitis scores from 0 to 4 were assigned based on the percent mononuclear infiltration of the islets: 0 = normal islet, 1 = trace (<10%), 2 = mild (10-30%), 3 = moderate (30-70%), or 4 = severe (>70%) infiltration. Pancreas sections were analyzed by a person unaware of the treatment status.

Immunofluorescence staining used mouse anti-glucagon (Sigma) and guinea pig anti-insulin (DAKO); secondary antibodies were Alexa-Fluor 488 and 592 goat anti-mouse and anti-guinea pig (Invitrogen). Images were captured using spinning-disk confocal microscopy (Nikon Eclipse TE2000-E) and analyzed with MetaMorph or NIS-Elements AR software. 

### Serum collection and ELISA analysis

Blood was collected with Trasylol (Sigma) at Day 1, 4, 7, and 11 following virus treatment. Serum was aliquoted and stored at -80°C until analysis by ELISA for IL-1β, IL-6, IFN-γ (Signosis), IL-12 (Invitrogen), MCP-1, haptoglobin and insulin (Alpco).

### Statistical analysis

Survival curves were computed by Kaplan-Meier with log-rank test; all other data were analyzed by ANOVA (GraphPad Prism 5.0) with Bonferroni post hoc test.

## Results

### Salicylate treatment prevents KRV+pIC induced type 1 diabetes in a dose-dependent manner

**Figure 1:**
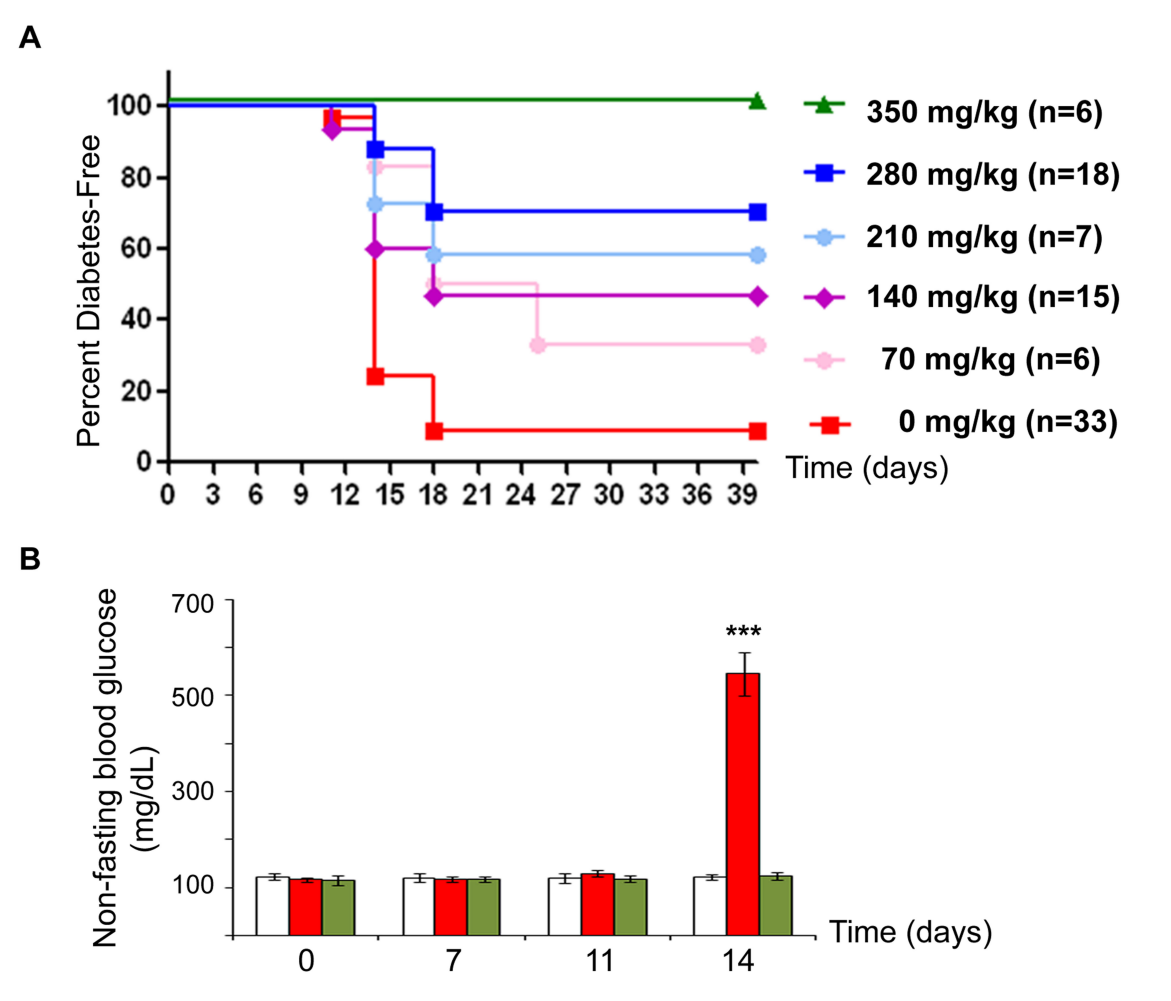
Sodium salicylate improves blood glucose levels and diabetes-free survival of KRV+pIC treated rats. BBDR rats were pre-treated with pIC and 0 to 350 mg/kg of sodium salicylate (SS) for 3 consecutive days (-3, -2, -1); KRV treatment and an additional treatment with SS was given on day 0. Doses of SS from 70 to 350 mg/kg body weight corresponded to 2- to 10-fold the amount given daily (for 14 weeks) to TINSAL-T2D patients [16]. (**A**) Kaplan-Meier curve showing onset of diabetes. As expected, >90% of rats given KRV+pIC without SS treatment became diabetic by 14-18 days, ***p<0.001. (**B**) Blood glucose levels were measured at the indicated times; n=6 for all groups, untreated (white), KRV+pIC (red), and KRV+pIC+SS (green); ***p<0.001, KRV+pIC *vs*. untreated and KRV+pIC+SS groups.

To determine the efficacy of salicylate to prevent virus-induced diabetes, we treated BBDR rats with KRV+pIC and a 5-fold dose range of sodium salicylate. Co-administration of 350 mg/kg salicylate prevented diabetes onset nearly completely, and the lowest dose tested (70 mg/kg) resulted in ~25% lower incidence of disease (Figure 1A). Interestingly, salicylate co-treated rats that developed T1D did so with similar kinetics (Figure 1A) and blood glucose levels (data not shown) as the KRV+pIC treated rats. Blood glucose levels of salicylate co-treated rats that remained diabetes-free were similar to control rats and significantly different from those of KRV+pIC treated rats (Figure 1B). All salicylate-treated rats that remained diabetes-free appeared healthy and gained weight similar to untreated controls (data not shown), indicating that no overt toxicity was associated with these doses of salicylate. 

To investigate whether the preventative effects of salicylate treatment was sustainable, a subset of the non-diabetic KRV+pIC+SS treated rats were monitored for an additional two to six months: all these rats (n=9) remained healthy and diabetes-free. In addition, we included a group of rats treated with pIC and a non-diabetogenic virus, H1. Although H1 and KRV have 98% sequence identity, H1+pIC treatment did not induce diabetes in this study (n=12), consistent with previous reports [7,18].

### Salicylate reduces serum levels of KRV+pIC induced proinflammatory cytokines

In order to assess proinflammatory cytokines during diabetes induction and their potential modulation by salicylate treatment, serum samples were collected at selected time points after KRV+pIC treatment in the highest dose groups (280 and 350 mg/kg) of salicylate. To distinguish the contribution of pIC in modulating the serum cytokine levels of KRV+pIC treated rats, we included a group treated with pIC only. BBDR rats (n=6) treated with this low dose of pIC did not develop diabetes, as we previously reported [18]. 

**Figure 2:**
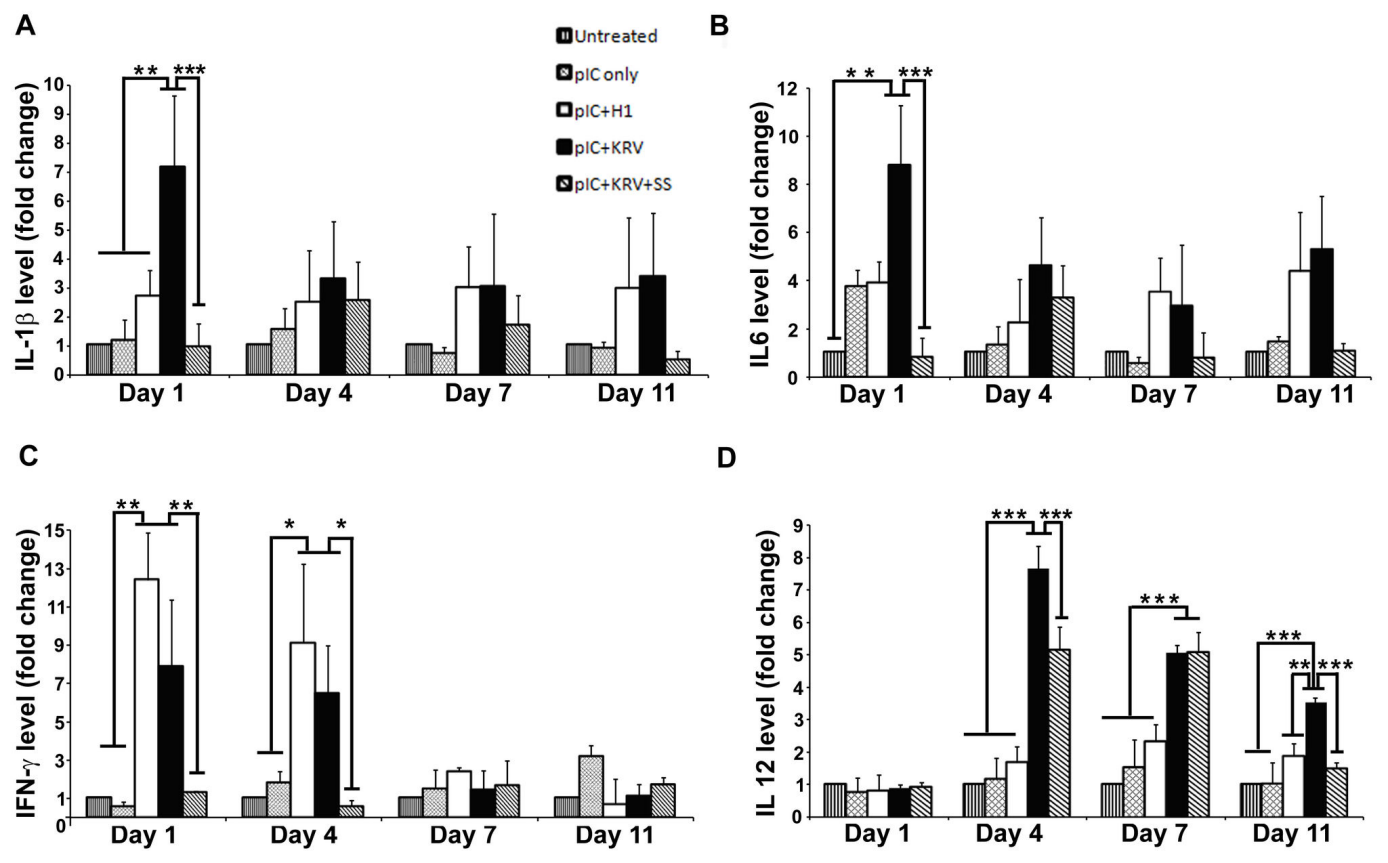
Proinfammatory cytokines are reduced in salicylate treated BBDR rats that remained diabetes-free. BBDR rats were untreated (vertical), pre-treated with pIC alone (hatched), H1+pIC (clear), KRV+pIC (black), or KRV+pIC+SS (diagonal). Serum samples were collected from rats at 1, 4, 7, and 11 days following KRV or H1 treatment and analyzed for (**A**) IL-1β. (**B**) IL-6, (**C**) IFN-γ, and (**D**) IL-12. Untreated control, n=3; mean values of control rats for IL-1β. IL-6, IFN-γ, and IL-12 were 1.85, 0.54, 2.07, and 0.47 ng/ml, respectively. For all treated groups, n≥6. Mean and standard error are shown; *p<0.05; **p<0.01; ***p<0.001.

KRV+pIC induced significant elevations of IL-1β, IL-6, and IFN-γ one day after treatment compared to untreated controls (Figure 2A-C). Yet, in each case, salicylate blocked the initial KRV+pIC induced elevations in IL-1β, IL-6, and IFN-γ almost completely. IL-1β and IL-6 levels in rats treated with non-diabetogenic H1+pIC were not significantly different from those of untreated controls, whereas IFN-γ levels were elevated similar to those of KRV+pIC treated rats. In general, serum levels of cytokines in pIC only treated rats were similar to those of untreated control rats, with the exception of IL-6, which was somewhat elevated at day 1, but less than KRV+pIC treated rats. At all time points analyzed, serum levels of the chemokine, MCP-1, were not statistically different between untreated and treated groups (data not shown). 

In contrast to the rapid induction of IL-1β, IL-6, and IFN-γ in response to KRV+pIC treatment, IL-12 had much slower kinetics (Figure 2D). There was no detectable increase in IL-12 at day 1 with KRV+pIC treatment, however, levels were elevated ~8-fold at day 4 and remained elevated at later time points when other cytokines had returned towards baseline levels. Importantly, both non-diabetogenic pIC and H1+pIC treatments failed to induce IL-12 at any time point analyzed, suggesting that IL-12 may be indicative of a diabetogenic inflammatory process. Although salicylate co-treatment did not completely block elevation of IL-12 in KRV+pIC treated rats, the levels were significantly ameliorated at days 4 and 11. 

### Salicylate inhibits KRV+pIC induction of the acute phase protein, haptoglobin

**Figure 3:**
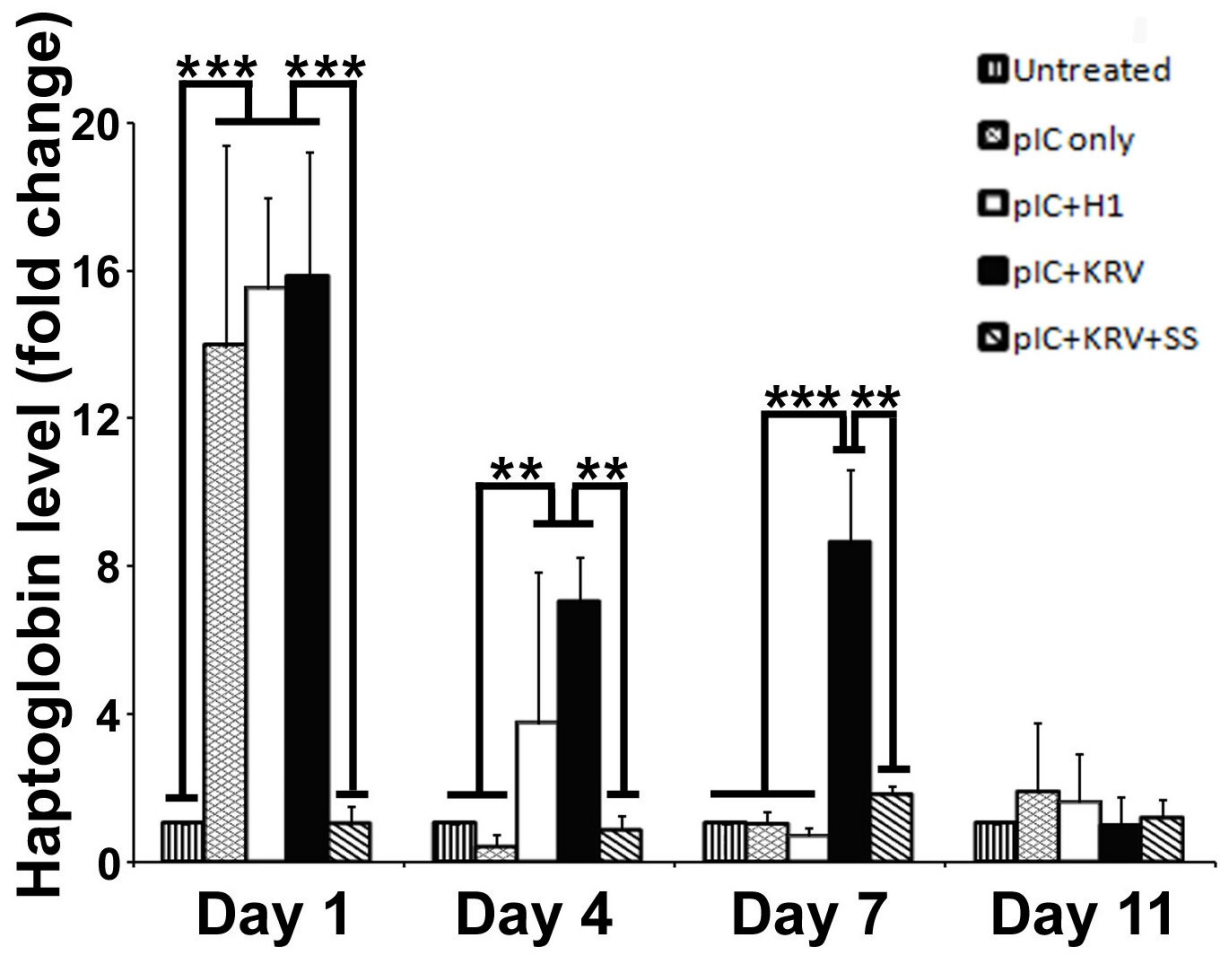
Salicylate blocks induction of the acute phase protein, haptoglobin. BBDR rats were untreated (vertical), pre-treated with pIC alone (hatched), H1+pIC (clear), KRV+pIC (black), or KRV+pIC+SS (diagonal). Serum samples were collected from rats at 1, 4, 7, and 11 days following KRV treatment and analyzed for haptoglobin. Untreated control, n=3; all treated groups, n≥6. Mean and standard error are shown; **p<0.01; ***p<0.001.

Using proteomic analyses we previously reported that serum levels of haptoglobin increased dramatically in BBDR rats treated with KRV+pIC [18]; here we confirm this finding by ELISA and further investigate the effect of salicylate to modulate haptoglobin levels. On day 1, KRV+pIC, H1+pIC, and pIC only treated rats had a ~15-fold increase in serum haptoglobin (Figure 3). Although haptoglobin levels in H1+pIC and pIC only treated rats subsequently declined, the levels in KRV+pIC treated rats remained elevated through day 7. At all time points analyzed, however, salicylate treatment completely blocked the KRV+pIC induced increase in serum haptoglobin. 

### Normal islet morphology and insulin staining in KRV+pIC and salicylate-treated rats that remain diabetes-free

**Figure 4:**
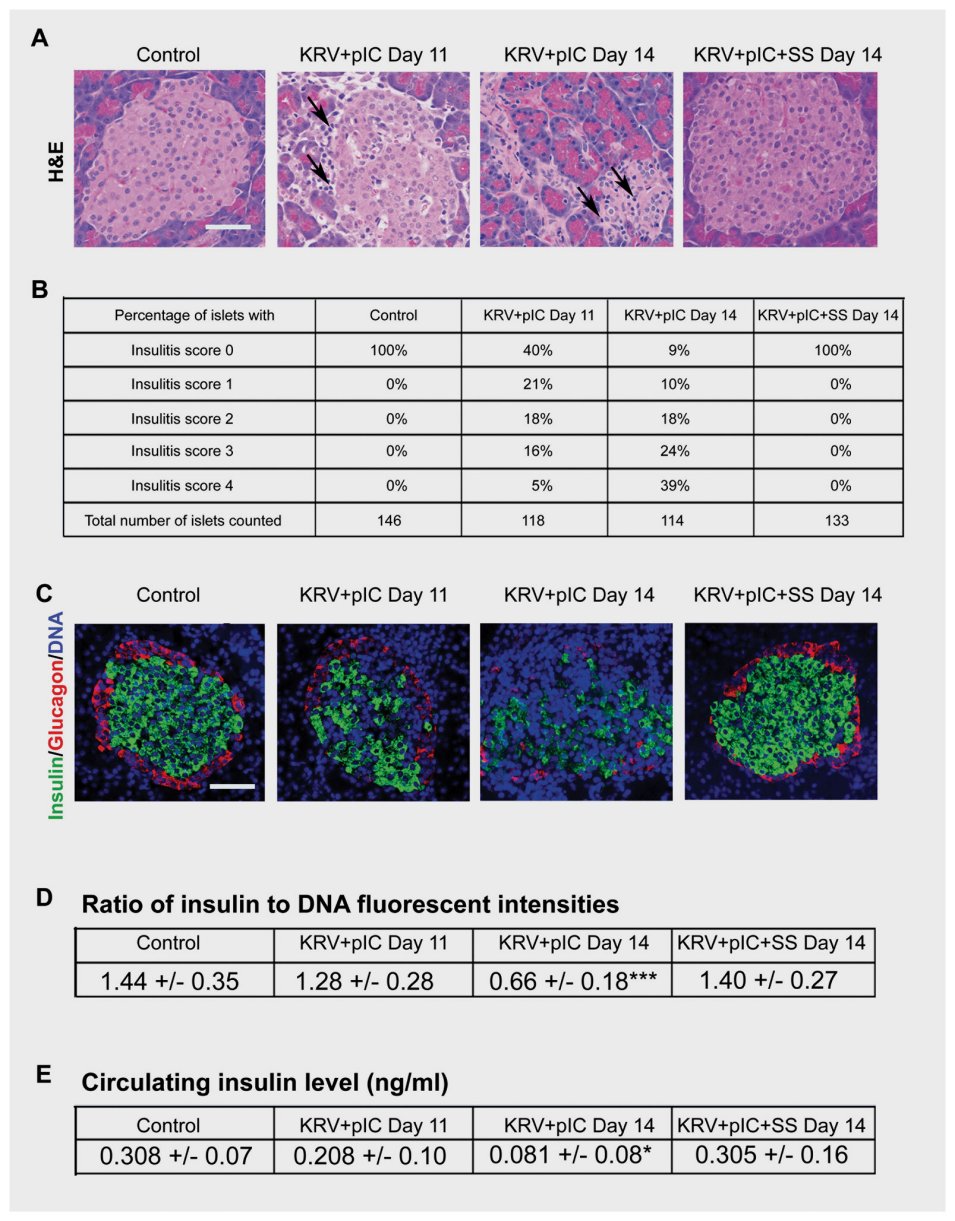
Insulitis and subsequent loss of islet and serum insulin are completely abrogated in salicylate treated BBDR rats that remained diabetes-free. BBDR rats were untreated or treated with KRV+pIC ± SS. (**A**) Pancreas samples were collected at the times indicated and stained for H&E (arrows indicate infiltrating lymphocytes). Panels shown are representative of n≥6 rats per group; scale bar, 50 µm. (**B**) Insulitis scores were determined from pancreas sections of 6-10 rats per group, 10-25 islets per rat. (**C**) Pancreas samples were collected and stained for insulin (green), glucagon (red), and DNA (DAPI, blue). Left panels are from an untreated (Control) BBDR rat, middle panels are from KRV+pIC treated rats at onset of insulitis (day 11) and hyperglycemia (day 14), and right panels are from a KRV+pIC+SS treated rat that was diabetes-free at day 14. Panels shown are representative of n≥3 rats per group; scale bar, 50 µm. (**D**) Insulin to DNA ratio is represented by green to blue intensity ratio; mean and standard error were calculated with n=9 islets per group, ***p<0.001, KRV+pIC *vs*. Control and KRV+pIC+SS groups. (**E**) Circulating serum levels of insulin were assayed at the indicated times; mean and standard error were calculated from n=6 rats per group, ***p<0.001, KRV+pIC *vs*. Control and KRV+pIC+SS groups.

To investigate the effect of salicylate treatment within the target organ, pancreata of treated rats were collected at selected time points and processed for morphology and insulin/glucagon staining. Consistent with earlier reports [19], lymphocytic infiltration of pancreatic islets (insulitis) of KRV+pIC treated rats was not detectable until day 11, with severe insulitis noted at day 14 (Figure 4A-B). In contrast, islets of all non-diabetic KRV+pIC+SS treated rats examined on day 14, regardless of SS dose, had no detectable insulitis and were morphologically indistinguishable from untreated age-matched control rats. Pancreata of BBDR rats treated with pIC only or H1+pIC had normal islet morphology and insulin staining at day 14 as well (data not shown). 

As expected, increasing severity of insulitis correlated with a progressive loss of insulin staining in KRV+pIC treated rats (Figure 4C-D). Concomitant with the loss of insulin staining in the islets, circulating levels of serum insulin also progressively declined (Figure 4E). In contrast, insulin staining of islets from non-diabetic KRV+pIC+SS treated rats was similar to control rats (Figure 4C-D), as were serum insulin levels (Figure 4E).

## Discussion

In this study we utilized the virus-inducible BBDR rat model to examine the effects of salicylate treatment to modulate type 1 diabetes progression. To our knowledge this study is the first to investigate the effects of NSAID on autoimmune type 1 diabetes pathogenesis. First, type 1 diabetes was prevented in a dose-dependent manner by treatment with the NSAID, salicylate. Second, we established distinct temporal profiles of selected inflammatory cytokines (IL-1β, IL-6, IL-12, and IFN-γ) and haptoglobin in the sera of BBDR rats in response to KRV+pIC treatment; significant elevations of IL-1β and IL-12, coupled with sustained elevations of haptoglobin, were specific to KRV+pIC and not found in rats co-treated with pIC and H1, a non-diabetogenic virus. Third, doses of salicylate that prevented diabetes also blocked or reduced elevations in these inflammatory cytokines and haptoglobin. Fourth, the pancreata of KRV+pIC+SS treated rats that remained diabetes-free showed normal islet morphology and insulin staining, and the islets were free of detectable insulitis. Fifth, SS treatment led to permanent protection from diabetes, even after withdrawal of the drug. Collectively, these data underscore the importance of the initial innate immune response in the pathogenesis of virus-inducible diabetes, and highlight the need to develop new diabetes therapies that target the inflammatory innate immune response.

Gene expression and genetic association studies support an emerging link between the innate immune response and susceptibility to human type 1 diabetes. An interferon regulatory factor 7-driven inflammatory network (IDIN) enriched for viral response genes has been identified in the BB rat [20], and genes from the analogous human IDIN have been shown to associate with susceptibility to type 1 diabetes [21]. In addition, a human genome-wide SNP scan identified the viral RNA receptor gene region IFIH1 (interferon induced with helicase C domain 1) as a type 1 diabetes susceptibility gene. Higher IFIH1 gene expression levels are found in the PBMCs of individuals with the susceptible genotype, and signaling through IFIH1 activates interferon-regulatory and other transcription factors (e.g., NF-κB) that, in turn, affect the levels and types of cytokines produced [22]. Our data indicate that elevations in IL-1β and IL-12 at the initial stages of KRV+pIC treatment, coupled with sustained elevations of haptoglobin, are associated with progression to diabetes in BBDR rats.

A recent functional genomics study of human sera found the importance of IL-1 in type 1 diabetes pathogenesis [23], supporting the relevance of our cytokine data obtained from the BBDR rat. Innate immune response and IL-1 regulated genes were identified in PBMCs incubated with sera from recent-onset type 1 diabetes patients, but not from sera of long-standing diabetes patients or healthy controls [23]. Moreover, even in pre-diabetic children, multiple proinflammatory cytokines, including IL-1β and IL-12, were significantly higher in children positive for islet autoantibodies compared to age-matched autoantibody-negative control subjects [24].

In our study, salicylate blocked or reduced the KRV+pIC induced elevations of serum proinflammatory cytokines and the acute phase protein, haptoglobin. Similarly, the acute phase protein, CRP, was reduced in type 2 diabetes patients given salsalate in the TINSAL-T2D study [16]. In both cases, salicylates likely prevent or ameliorate diabetes by reducing the magnitude of the inflammatory innate immune response. Speculatively, ‘hyperactivation’ of the innate immune system may reach a threshold above which, in genetically-susceptible individuals, an islet-specific adaptive (auto) immune response is engaged that progresses to type 1 diabetes. As such, in those patients at-risk for type 1 diabetes, we envision that ‘preventative’ treatment may only require salicylate treatment transiently, when patients have evidence of an infection (e.g., fever). In this case, salicylate could be given short term (e.g., several days) to dampen the innate inflammatory response, yet still allow for clearance of the virus/pathogen. 

In summary, the BBDR model is a powerful tool to elucidate the causes and consequences in virus-inducible autoimmune diabetes. The similar serum cytokines in human type 1 diabetes and virus-induced BBDR rats support the use of this model for identifying cellular and molecular mechanisms involved, as well as pre-clinical testing of type 1 diabetes therapeutics for safety and efficacy. Our novel discovery provides insight into the potential use of salicylate as a stand-alone or combination therapy to prevent and/or treat human type 1 diabetes. Given the well-known safety record for salicylate, its use in clinical trials of pre-diabetic or recent-onset patients may be warranted.
